# Deep Learning in mHealth for Cardiovascular Disease, Diabetes, and Cancer: Systematic Review

**DOI:** 10.2196/32344

**Published:** 2022-04-04

**Authors:** Andreas Triantafyllidis, Haridimos Kondylakis, Dimitrios Katehakis, Angelina Kouroubali, Lefteris Koumakis, Kostas Marias, Anastasios Alexiadis, Konstantinos Votis, Dimitrios Tzovaras

**Affiliations:** 1 Information Technologies Institute Centre for Research and Technology Hellas Thessaloniki Greece; 2 Institute of Computer Science Foundation for Research and Technology Hellas Heraklion Greece

**Keywords:** mHealth, deep learning, chronic disease, review, mobile phone

## Abstract

**Background:**

Major chronic diseases such as cardiovascular disease (CVD), diabetes, and cancer impose a significant burden on people and health care systems around the globe. Recently, deep learning (DL) has shown great potential for the development of intelligent mobile health (mHealth) interventions for chronic diseases that could revolutionize the delivery of health care anytime, anywhere.

**Objective:**

The aim of this study is to present a systematic review of studies that have used DL based on mHealth data for the diagnosis, prognosis, management, and treatment of major chronic diseases and advance our understanding of the progress made in this rapidly developing field.

**Methods:**

A search was conducted on the bibliographic databases Scopus and PubMed to identify papers with a focus on the deployment of DL algorithms that used data captured from mobile devices (eg, smartphones, smartwatches, and other wearable devices) targeting CVD, diabetes, or cancer. The identified studies were synthesized according to the target disease, the number of enrolled participants and their age, and the study period as well as the DL algorithm used, the main DL outcome, the data set used, the features selected, and the achieved performance.

**Results:**

In total, 20 studies were included in the review. A total of 35% (7/20) of DL studies targeted CVD, 45% (9/20) of studies targeted diabetes, and 20% (4/20) of studies targeted cancer. The most common DL outcome was the diagnosis of the patient’s condition for the CVD studies, prediction of blood glucose levels for the studies in diabetes, and early detection of cancer. Most of the DL algorithms used were convolutional neural networks in studies on CVD and cancer and recurrent neural networks in studies on diabetes. The performance of DL was found overall to be satisfactory, reaching >84% accuracy in most studies. In comparison with classic machine learning approaches, DL was found to achieve better performance in almost all studies that reported such comparison outcomes. Most of the studies did not provide details on the explainability of DL outcomes.

**Conclusions:**

The use of DL can facilitate the diagnosis, management, and treatment of major chronic diseases by harnessing mHealth data. Prospective studies are now required to demonstrate the value of applied DL in real-life mHealth tools and interventions.

## Introduction

### Background

Chronic, noncommunicable diseases are the leading cause of mortality and disability worldwide. According to the World Health Organization, cardiovascular disease (CVD) is the number 1 cause of death worldwide, taking an estimated 17.9 million lives each year [1]. In 2020, there were approximately 10 million deaths because of cancer [2]. Diabetes is another major chronic disease, with the number of people diagnosed with it increasing dramatically from 108 million in 1980 to 422 million in 2014 [3]. As a consequence of the prevalence of chronic diseases, health care systems around the globe struggle to provide efficient medical care to those patients.

Mobile health (mHealth) has recently emerged as a new paradigm for providing efficient medical care anytime, anywhere. The wide uptake of mobile phones or other mobile electronic communication devices by people has fueled the advancement of their capabilities. Nowadays, mobile devices such as smartphones, smartwatches, and wearable devices can enable robust sensing and processing of health parameters along with communication of health information to patients and caregivers. As a result, they reinforce better daily self-management of chronic diseases by the patients themselves [4] and facilitate remote medical management [5]. In this light, the value of mHealth for chronic diseases has been depicted in several research works [6].

The regular use of mHealth devices around the clock has allowed for the generation of large data sets that can be harnessed by data analytics frameworks toward developing more intelligent mHealth interventions able to identify a range of medical risk factors, improve clinical decision-making, and revolutionize the delivery of health care services [7,8]. The challenge is that the sets of data captured by mHealth devices (eg, sensed data) are often too complex, unstructured, and heterogeneous, thereby creating obstacles in their processing and interpretation through traditional data mining and statistical learning approaches. Deep learning (DL), which is founded on artificial neural networks, appears as a key technology for providing suitable algorithmic frameworks in this direction [9]. DL allows computational models that are composed of multiple processing layers to learn representations of data with multiple levels of abstraction and requires little engineering by hand [10]. DL models have demonstrated great potential in different domains of health care and have shown excellent performance in computer vision, natural language processing, and mining of electronic health records as well as mHealth modalities and sensor data analytics [11].

### Objectives

Despite the potential of DL for mHealth, there have not been targeted reviews in this field. Other reviews have been broad [8,12], not closely related to mHealth [11,13], or not focused on major chronic diseases with the largest prevalence worldwide [14]. In this context, the aim of this paper is to provide a systematic review of the currently available literature and identify recent studies that have used DL based on mHealth data for the diagnosis, prognosis, management, and treatment of major chronic diseases (ie, CVD, diabetes, and cancer). Our ultimate goal is to advance the understanding of researchers, caregivers, and engineers of the progress made in this rapidly developing field.

## Methods

### Search Strategy

A search was conducted on the web-based bibliographic databases Scopus and PubMed in March 2021 to identify studies published during the last 10 years that incorporated DL in the context of mHealth for CVD, diabetes, and cancer.

### Eligibility Criteria

The inclusion criteria for study selection were as follows: (1) DL algorithm or algorithms should be used and quantitative outcomes in terms of their performance should be presented in the study; (2) the DL algorithm in the study should harness mHealth data acquired through a mobile or wearable device; (3) the study should focus on the diagnosis, prognosis, management, or treatment of one of the major chronic diseases with the largest prevalence worldwide (CVD, diabetes, or cancer); and (4) the paper describing the study must have been published in English. Case reports, letters to editors, preprint papers, qualitative studies, surveys or reviews, simulation studies, and studies describing protocols were excluded from the review.

### Study Selection

The following string—*(deep learning) OR (neural networks) AND (mobile health) OR (smartphone) OR (mobile phone) OR (mobile device) OR (mobile app) OR (smartwatch) OR (wearable) OR (sensor) AND (health)*—was used for searching within the title, abstract, and keywords of the manuscripts. The retrieved records from Scopus and PubMed were imported into the Mendeley (Mendeley Ltd) bibliography management software to identify duplicates. Authors AT, HK, DK, AK, LK, and AA independently screened the papers that were obtained as a result of the aforementioned search string to minimize bias in the selection process and reduce possible errors. In case of disagreements, these were resolved through discussion between the authors to reach a consensus. The screening procedure took place in 2 stages. In the first stage, the abstracts of the candidate papers for inclusion were screened by the authors according to the defined inclusion and exclusion criteria. In the second stage, the authors read the full manuscripts of the eligible papers, as identified in the first stage, and selected the final papers for inclusion.

The included studies were synthesized by the authors according to the target disease, the number of enrolled participants and their age, and the study period as well as the DL algorithm used, the main outcome of the algorithm, the data set used, the features selected, and the achieved performance. This systematic review was conducted following the PRISMA (Preferred Reporting Items for Systematic Reviews and Meta-Analyses) guidelines [15]. A completed PRISMA checklist is shown in Multimedia Appendix 1 [16].

## Results

### Overview

**Figure 1:**
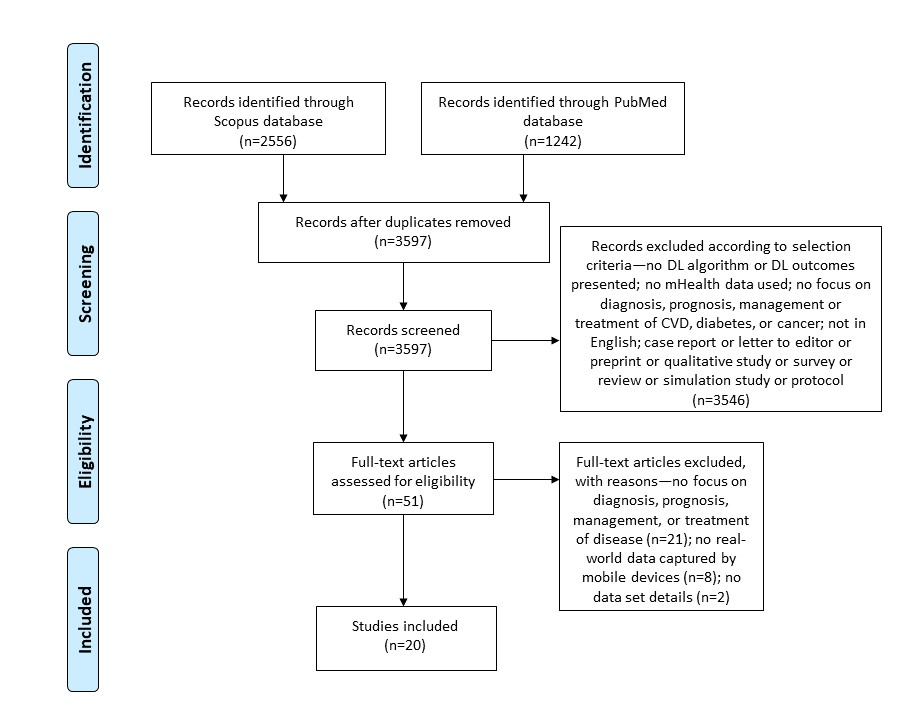
PRISMA (Preferred Reporting Items for Systematic Reviews and Meta-Analyses) flow diagram. CVD: cardiovascular disease; DL: deep learning; mHealth: mobile health.

The literature search resulted in 2556 articles from Scopus and 1242 articles from PubMed (3798 articles in total). A total of 94.71% (3597/3798) of records were screened after the removal of 5.29% (201/3798) duplicates. Of those 3597 articles, 3546 (98.58%) were excluded because they did not meet the eligibility criteria. After reading the full texts of the remaining 51 articles, the number of eligible articles was reduced to 20 (39%). Reasons for the exclusion of articles are shown in Figure 1.

### Applications of DL and Outcomes

#### Overview

Table 1 shows the primary characteristics of the included studies in terms of target disease, number of participants, and their age as well as study duration (where applicable). Of the 20 DL studies, 7 (35%) targeted CVDs, 9 (45%) targeted diabetes, and the remaining 4 (20%) targeted cancer. An interesting finding is that the number of participants included in the DL studies for diabetes was small (range 6-46) compared with CVD (range 10-70,000) and cancer (range 99-917).

**Table 1:** Characteristics of the included studies (N=20).

Study	Target disease	Participants, N	Age	Study period
Al-Makhadmeh et al [17]	CVD^a^	10	N/A^b^	No
Ali et al [18]	CVD	597 (2 data sets combined with 303 and 294 participants)	29-79 years	No
Dami et al [19]	CVD	Four databases: (1) 70,000 participants, (2) 20,000 participants, (3) 139 patients with hypertension, and (4) 303 participants	N/A	Participants in database 3 were followed for 12 months
Deperlioglu et al [20]	CVD	N/A	N/A	Usability study for 4 months
Fu et al [21]	CVD	20,000	N/A	No (tested in the real world)
Huda et al [22]	CVD	47	N/A	No
Torres-Soto et al [23]	CVD	163	Mean 68 (cardioversion cohort), 56 (exercise stress test cohort), and 67 (ambulatory cohort) years	No
Cappon et al [24]	Diabetes (T1DM^c^)	6	20-80 years	8 weeks
Chen et al [25]	Diabetes (T1DM)	6	20-80 years	8 weeks
Efat et al [26]	Diabetes	25	N/A	Data collected during a 2-month period
Faruqui et al [27]	Diabetes (T2DM^d^)	10 patients in the smartphone group (overweight or obese)	21-75 years	6 months
Goyal et al [28]	Diabetes	30	N/A	No (tested in the real world)
Joshi et al [29]	Diabetes	46	17-80 years	No
Sánchez-Delacruz et al [30]	Diabetes	15	29-62 years	No
Sevil et al [31]	Diabetes	25	Mean 24.88 (SD 3.15) years	430-hour experiment
Suriyal et al [32]	Diabetes	N/A	N/A	No
Ech-Cherif et al [33]	Cancer	N/A	N/A	No
Guo et al [34]	Cancer	N/A	N/A	No
Hu et al [35]	Cancer	917	N/A	No
Uthoff et al [36]	Cancer	99	Mean 40 (SD 14.1) years	No (tested in the real world)

^a^CVD: cardiovascular disease.

^b^N/A: not applicable.

^c^T1DM: type 1 diabetes mellitus.

^d^T2DM: type 2 diabetes mellitus.

Only 25% (5/20) of the studies reported the integration of their developed model into a DL-empowered system or application that was tested in real life [20,21,28,33,36]. However, none of the studies presented a clinical validation of the deployed systems and applications (eg, through randomized controlled trials).

Approximately 25% (5/20) of the DL studies used an unseen external data set for evaluation purposes to eliminate possible bias and build models that could be generalized [20,23,33,36,37]. Different performance outcomes were reported in the identified studies using DL algorithms (Table 2). None of the included studies used any guidelines for reporting the development and outcomes of the models such as TRIPOD (Transparent Reporting of a Multivariable Prediction Model for Individual Prognosis or Diagnosis) [38]. All the included studies (20/20, 100%) are briefly described below in terms of purpose, data and algorithms used, and evaluation outcomes.

**Table 2:** Algorithms and outcomes of the included studies (N=20).

Study	DL^a^ outcome	DL algorithm	Data used	Features selected	Performance	Comparison with classic ML^b^ algorithms
Al-Makhadmeh et al [17]	Detection of heart disease	Higher-order Boltzmann deep belief neural network	123 instances and 23 attributes collected from 10 patients using sensor devices from data sets available in UCI^c^ repository	ECG^d^, blood pressure, chest pain typology, cholesterol level, vessel information, minimum and maximum heart rate, angina, and depression symptoms	99.5% sensitivity	No
Ali et al [18]	Detection of heart disease	Feedforward network that uses backpropagation techniques and gradient algorithms (ensemble approach)	Cleveland and Hungarian data sets available from UCI repository containing EMR^e^ and sensor data (physiological measurements)	Demographic (age and sex), clinical (chest pain type, number of major vessels colored by fluoroscopy, and exercise test results), and sensor (resting blood pressure and fasting blood sugar)	84% accuracy	SVM^f^ (71.8%), logistic regression (73.7%), random forest (73.7%), decision tree (74.8%), and naïve Bayes (80.4%)
Dami et al [19]	Prediction of cardiovascular events to prevent SCD^g^ and heart attacks	Combination of deep belief network and LSTM^h^ RNN^i^	Four databases: (1) Kaggle heart disease data set archive, (2) database from Shahid Beheshti Hospital Research Center, (3) database from PhysioNet site including patients from the Naples Federico II University Hospital in Italy, and (4) UCI4 data set Archive from 1988	Age, sex, weight, height, body surface area, BMI, smoker or not, systolic blood pressure, diastolic blood pressure, intima media thickness, left ventricular mass index, and ejection fraction	88% accuracy, 87% F-measure, and 87% precision	Logistic regression, SVM, and random forest (56% accuracy on average)
Deperlioglu et al [20]	Classification of heart sounds	Autoencoder neural networks	PASCAL^j^ B-training heart sound data sets and A-training heart sound data sets	No	96.03% accuracy for normal diagnosis, 91.91% accuracy for extrasystole diagnosis, and 90.11% accuracy for murmur diagnosis	SVM, naïve Bayes, decision tree, and AdaBoost (84.2%-93.3% accuracy)
Fu et al [21]	CVD^k^ detection	A hybrid of a CNN^l^ and an RNN	The test set includes 15,437 anonymous ECG recordings collected from several tertiary hospitals in China	No	95.53%-99.97% accuracy of CVD	No
Huda et al [22]	Arrhythmia detection	CNN	MIT-BIH^m^ arrhythmia data set obtained from PhysioNet	No	94.03% accuracy in classifying abnormal cardiac rhythm	No
Torres-Soto et al [23]	Arrhythmia event detection	Pretraining using convolutional denoising autoencoders followed by CNN, transfer learning, and auxiliary signal quality estimation	Data available through synapse (Synapse ID: syn21985690)	Regions of the upslope from the systolic phase to be informative for AF^n^ class-specific predictions	98% sensitivity, 99% specificity, and 96% F_1_	Random forest (32% sensitivity, 79% specificity, and 39% F_1_)
Cappon et al [24]	Prediction of short-time blood glucose levels	LSTM RNN	OhioT1DM data set containing CGM^o^ data, lifestyle data (diet, exercise, and sleep), galvanic skin response, skin temperature, and magnitude of acceleration	CGM, injected insulin as reported by the pump, and self-reported meals and exercise	RMSE^p^ 20.20 and 34.19 for 30- and 60-minute prediction, respectively	No
Chen et al [25]	Prediction of short-time blood glucose levels	Dilated RNNs	The OhioT1DM data set of continuous glucose monitoring data and the corresponding daily events from 6 patients with type 1 diabetes	CGM, insulin doses, carbohydrate intake, and time index; additional data included exercise, heart rate, and skin temperature	15.299 to 22.710 RMSE for different participants	No
Efat et al [26]	Risk level classification of patients with diabetes	Artificial neural network	2-month data from 25 patients with diabetes	Patients’ age, sex, sugar level, heart pulse, food intake, sleep time, and exercise or calorie burn	84.29% accuracy, 82.35% sensitivity, and 86.11% specificity	No
Faruqui et al [27]	Prediction of daily glucose levels	LSTM RNN	10 patients with diabetes (T2DM^q^) being overweight or obese	Daily mobile health lifestyle data on diet, physical activity, weight, and previous glucose levels from the day before	33.33% (patient 7) to 86.67% (patient 2) accuracy	KNN^r^ regression (10%-56% accuracy)
Goyal et al [28]	Real-time DFU^s^ localization	Faster R-CNN^t^	Transfer learning with ImageNet (Stanford Vision Lab) and Microsoft COCO data set; 1775 images of DFUs	Low-level features such as edge detection, corner detection, texture descriptors, shape-based descriptors, and color descriptors	91.8% mean average precision	SVM (70.3% precision)
Joshi et al [29]	Continuous blood glucose monitoring	LMBP^u^	NIR^v^ optical spectroscopy data	No	AvgE^w^ 6.09%, mARD^x^ 6.07%	Multiple polynomial regression—AvgE and mARD were 4.88% and 4.86% for serum glucose examination
Sánchez-Delacruz et al [30]	Diabetic neuropathy detection	Classifiers combined with multilayer perceptron	Raw data from 5 accelerometers	Accelerometer data	85% accuracy	No
Sevil et al [31]	Classification of activity into 5 stages for determining the energy expenditure for diabetes therapy	RNN	Data sets not available	23 selected information features are reported in the paper out of 2216	94.8% classification accuracy	KNN, SVM, naïve Bayes, decision tree, linear discrimination, and ensemble learning (75.7% to 93.1% accuracy)
Suriyal et al [32]	Diabetic retinopathy detection	MobileNets in TensorFlow with the help of RMSprop and asynchronous gradient descent	Data set available in Kaggle database	No	73% accuracy, 74% sensitivity, and 63% specificity	No
Ech-Cherif et al [33]	Benign and malignant cancer detection	Resource-constrained, mobile-ready deep neural network	Three databases: DermNet, ISIC^y^ Archive, and Dermofit Image Library	Cancerous or not	91.33% accuracy	No
Guo et al [34]	Identification of cervix and noncervix images	Ensemble method that consists of 3 DL architectures: RetinaNet, deep SVDD^z^, and a customized CNN	Four data sets were used in this study: MobileODT, Kaggle, and COCO2017 for training and validation, and SEVIA^aa^ for testing	Normal samples as cervix images and from the anomalous samples as noncervix images	91.6% accuracy and 89% F_1_ score	No
Hu et al [35]	Detection of cervical precancer	Automated visual evaluation, RetinaNet, and Adam optimization algorithm	Microsoft COCO images, 7334 training images, 970 validation images, and 1058 test images	No specific features were reported in the paper	ROC^ab^ curve (AUC^ac^) of 0.95	No
Uthoff et al [36]	Early detection of precancerous and cancerous lesions in the oral cavity	CNN, VGG-M^ad^ network pretrained on the ImageNet data set	170 image pairs	WLI^ae^ and AFI^af^ provided the most information about type of lesion and size of the affected area	Sensitivity, specificity, positive predictive values, and negative predictive values (81.25%-94.94%); 0.908 AUC	No

^a^DL: deep learning.

^b^ML: machine learning.

^c^UCI: University of California, Irvine.

^d^ECG: electrocardiogram.

^e^EMR: electronic medical record.

^f^SVM: support vector machine.

^g^SCD: sudden cardiac death.

^h^LSTM: long short-term memory.

^i^RNN: recurrent neural network.

^j^PASCAL: Pattern Analysis, Statistical Modeling, and Computational Learning.

^k^CVD: cardiovascular disease.

^l^CNN: convolutional neural network.

^m^MIT-BIH: Massachusetts Institute of Technology–Beth Israel Hospital.

^n^AF: atrial fibrillation.

^o^CGM: continuous glucose monitoring.

^p^RMSE: root mean squared error.

^q^T2DM: type 2 diabetes mellitus.

^r^KNN: k-nearest neighbor.

^s^DFU: diabetic foot ulcer.

^t^R-CNN: region-based convolutional neural network.

^u^LMBP: Levenberg–Marquardt Backpropagation.

^v^NIR: near-infrared.

^w^AvgE: average error.

^x^mARD: mean absolute relative difference.

^y^ISIC: International Skin Imaging Collaboration.

^z^SVDD: support vector data description.

^aa^SEVIA: smartphone-enhanced visual inspection with acetic acid.

^ab^ROC: receiver operating characteristic.

^ac^AUC: area under the curve.

^ad^VGG-M: visual geometry group multi-scale.

^ae^WLI: white-light imaging.

^af^AFI: autofluorescence imaging.

#### CVD Studies

Huda et al [22] introduced a low-cost, low-power, and wireless electrocardiogram (ECG) monitoring system with DL-based automatic arrhythmia detection. The model was based on a 1D convolutional neural network (CNN) that provided an accuracy of 94.03% in classifying abnormal cardiac rhythm on the Massachusetts Institute of Technology–Beth Israel Hospital Arrhythmia Database.

Deperlioglu et al [20] described a secure Internet of Health Things system to provide real-time support to physicians for the diagnosis of CVDs. Heart sounds were classified using autoencoder neural networks (AENs), and the developed solution demonstrated better results than those reported in the literature studied.

In the study by Ali et al [18], a DL-based ensemble model was used for the detection of heart disease in 597 patients. More specifically, a feedforward neural network was used to perform binary classification of the presence or absence of disease. An 84% accuracy in this classification task was achieved by using publicly available data sets containing sensed data in terms of physiological measurements (such as blood pressure and fasting blood sugar) as well as electronic health record data (including exercise test results, chest pain information, and demographic information).

In the study by Al-Makhadmeh et al [17], the authors proposed the use of a Boltzmann deep belief model to detect whether a patient has heart disease. The model was based on data acquired from 10 patients (publicly available data set), including ECG and blood pressure measurements as well as other diagnostic information such as chest pain and appearance of angina or depression symptoms. A sensitivity of 99.5% was achieved.

Another approach to shed light on the occurrence of arterial and cardiovascular events was examined in the study by Dami et al [19]. A long short-term memory (LSTM) neural network and a deep belief network were used to predict arterial events over the course of a few weeks before the event using ECG recordings and time-frequency features of ECG signals. The proposed LSTM and deep belief network approach had significantly better performance when compared with all other DL approaches and traditional classifications.

Furthermore, in the study by Torres-Soto et al [23], the authors developed DeepBeat, a multitask DL method to detect arrhythmia events for atrial fibrillation in real time using wrist-based photoplethysmography devices. The proposed approach exploited transfer learning, and the resulting models had a sensitivity of 0.98, specificity of 0.99, and *F*_1_ score of 0.93.

In the study by Fu et al [21], an Internet of Things and cloud service system was designed that collected high-quality ECG data and diagnosed 20 types of CVDs using a DL model that was a hybrid between a CNN and a recurrent neural network. The model achieved >0.98 area under the receiver operating characteristic curve score on 17 of the diagnostic items.

#### Diabetes

For diabetes, there were also several approaches showing impressive performance using DL. For example, in the study by Sevil et al [31], the authors proposed DL with LSTM to determine physical activity states for use in automated insulin delivery systems. The approach exploited a multi-sensor wristband and achieved 94.8% classification accuracy.

In another approach by Suriyal et al [32], DL was used for the detection of diabetic retinopathy using mobile devices for real-time screening without requiring an internet connection. The approach exploited a TensorFlow deep neural network, with a reported accuracy of 73%.

Goyal et al [28] proposed an automated method for the detection and localization of diabetic foot ulcers (DFUs) based on images. The model was robust enough, with a mean average precision of 91.8%, and the trained model could run on simple hardware with a speed of 48 milliseconds for inferencing a single image and with a model size of 57.2 MB. The model was based on transfer learning that was initially trained with ImageNet (Stanford Vision Lab) and Microsoft COCO data sets and with DFU images in the final step. The authors also deployed these models on an Android phone to create real-time object localization for DFUs.

Joshi et al [29] proposed a wearable consumer device called iGLU 2.0, which was based on a DL model for glucose level prediction as a noninvasive, precise, and cost-effective solution to monitor blood glucose levels and control diabetes. The proposed glucometer used the concept of short-wave, near-infrared spectroscopy to predict blood glucose levels. The results were comparable with those of the serum glucose examination, an invasive laboratory examination.

A glucose prediction model was also developed in the study by Chen et al [25]. The authors used a new DL technique based on a dilated recurrent neural network model to predict future glucose levels for a prediction horizon of 30 minutes. Using this model, it was shown that the accuracy of short-time glucose predictions could be significantly improved.

The study by Efat et al [26] introduced a smart health monitoring tool for patients with diabetes. The objective of the authors was to use continuous sensor monitoring and processing with neural networks to provide a continuous evaluation of the patient health risk status.

In the study by Cappon et al [24], an LSTM model for the prediction of blood glucose concentration in patients with type 1 diabetes was proposed. The applied model was based on continuous glucose monitoring data collected from 6 patients as well as insulin dose and self-reported meals and exercise. A root mean squared error of 20.20 for prediction of glucose over the next 30 minutes and of 34.19 for prediction over the next hour was highlighted as the performance outcome of their work.

In the study by Faruqui et al [27], the authors used a DL model based on LSTM and developed a transfer learning strategy (to cope with data scarcity and improve the model’s personalization capabilities) to dynamically forecast daily glucose levels. The patient data used for their model were the daily mHealth lifestyle data and the glucose levels from the day before. The model achieved considerable accuracy in predicting the next day glucose level based on the Clark Error Grid and –10% to +10% range of the actual values on data collected from 10 patients who had been monitored daily for over 6 months.

In the study by Sánchez-Delacruz et al [30], the detection of diabetic neuropathy through the application of a multilayer perceptron combined with additional classifiers on raw accelerometer data was proposed. A total of 15 individuals (10 with diabetic neuropathy and 5 healthy) wearing 5 accelerometers were instructed to walk. The algorithm was able to reach 85% accuracy in diabetic neuropathy recognition.

#### Cancer

Several studies also focused on cancer. In the study by Hu et al [35], the authors exploited a new DL algorithm called automated visual evaluation for analyzing cervigram images captured by commodity mobile phones to detect cervical precancer. This approach achieved a receiver operating characteristic curve (area under the curve) of 0.95.

In another approach by Uthoff et al [36], the authors used a CNN to enable early detection of precancerous and cancerous lesions in the oral cavity with the potential to reduce morbidity, mortality, and health care costs. To achieve this, the authors used a custom Android app that synchronized an external light-emitting diode and image capture for autofluorescence imaging and white-light imaging on a smartphone. The sensitivity, specificity, positive predictive value, and negative predictive value of the approach ranged from 81.25% to 94.94%.

DL techniques have also been applied for triaging skin cancer detection. The authors in the study by Ech-Cherif et al [33] manually trained a resource-constrained deep CNN called MobileNetV2 to identify the binary classification of skin lesions using benign and malignant as the 2 classes. When the model was tested on an unseen library of images using an iOS mobile app, it was found that all images were correctly classified.

In the study by Guo et al [34], the authors combined the assessment of 3 DL architectures to determine whether an image contained a cervix. The study showed that the ensemble method outperformed individual DL methods. Such data quality algorithms could be used to clean large data sets and provide quality assurance for machine learning (ML) algorithms in routine clinical use.

### Architectures of DL Models

DL approaches in mHealth can be efficient by taking advantage of the large volumes of data generated through the use of mobile and sensing devices. In Table 3, we provide details regarding the DL architectures and parameters or hyperparameters used in the selected studies to shed light on the most promising ones used in practice. It is apparent that there is no single best DL architecture to be used for mHealth considering that the selection of the most appropriate DL architecture is mainly data driven.

Regarding the hyperparameters of the studied models, the layers varied between 3 and 50. In most cases, softmax or sigmoid activation functions were used and applied primarily to classification problems, the losses L1 and L2 were <0.01 and, in some cases, Adam optimization was used.

Huda et al [22] proposed a CNN model architecture that consisted of 1D convolution, max-pooling, batch normalization, and dropout layers. The flattened layer output was passed through a fully connected layer with dropout and a second fully connected dense layer. A softmax layer with 14 outputs was then used for arrhythmia classification.

Torres-Soto et al [23] focused on detecting arrhythmia events using unsupervised transfer learning through convolutional denoising autoencoders (CDAEs). The authors applied a 2-stage training to address the unbalanced data problem common to biomedical applications, exploiting a multitask CNN architecture, transfer learning, and an auxiliary signal quality estimation task for atrial fibrillation event detection from spatially segmented physiological photoplethysmography signals. Unsupervised pretraining was performed using CDAEs. The authors then used convolutional and pooling layers in the encoder and upsampling and convolutional layers in the decoder. To obtain the optimal weights, they were randomly initiated according to the He distribution, and the gradient was calculated using the chain rule to backpropagate error derivatives through the decoder network and then through the encoder network. Using a number of hidden units lower than the inputs forces the autoencoder to learn a compressed approximation. The loss function used in pretraining was the mean squared error and was optimized using a backpropagation algorithm. Finally, 3 convolutional layers and 3 pooling layers were used for the encoder segment, and 3 convolutional layers and 3 upsampling layers were used for the decoder segment of the CDAE. A Rectified Linear Unit (ReLU) was applied as the activation function, and Adam was used as the optimization method. Each model was trained with mean squared error loss for 200 epochs, with a reduction in learning rate of 0.001 for every 25 epochs if the validation loss did not improve.

For cervical precancer detection using a smartphone [35], a Resnet-50 architecture was proposed. The whole process started with image augmentation methods (random image scale, random horizontal or vertical flip, random rotation, random shearing, random translation, and transforming the red channel of the image through a *γ* transformation with *γ* randomly chosen). Nonmaximum suppression after processing was then followed after cervix or precancerous cervix object detection. The model parameters were initialized with weights pretrained on Microsoft COCO images. All model parameters were then fine-tuned using the visual inspection with acetic acid training data. For the optimization strategy, the authors used the Adam optimization algorithm, fixing the clipnorm parameter at the default of 0.001, and they also used a learning rate of 1 × 10^–5^. The metrics used for hyperparameter (number of iterations and batch size) optimization were the mean average precision and validation classification loss.

For smartphone-based oral cancer screening [36], classification using a CNN was applied. For the CNN training, methods commonly used in network training were used, including transfer learning and data augmentation. For data augmentation, the original images were rotated and flipped to feed the network with more training data. In addition, transfer learning was applied using a visual geometry group multi-scale network pretrained on the ImageNet data set. The network was modified for the task by replacing the final dense layer and softmax layer and then training the network with the available data set.

Goyal et al [28] used transfer learning from massive data sets in nonmedical backgrounds such as ImageNet and Microsoft COCO data sets for the initial training of their image model for DFU localization. The authors used two CNNs, MobileNet and Inception-V2, and set the weight for L2 regularizer as 0.00004 and batch normalization with a decay of 0.9997 and epsilon of 0.001. A batch size of 24 was used along with the optimizer as RMSprop with a learning rate of 0.004 and decay factor of 0.95. The momentum optimizer value was set at 0.9 with a decay of 0.9 and epsilon of 0.1.

The DL approach was used for physical activity classification for automated insulin delivery systems [31], combining different layers including fully connected, LSTM, softmax, regression, ReLU, and dropout layers. In addition, the authors used the L2 regularization term to reduce the risk of overfitting (value 0.05).

In another approach, a TensorFlow deep neural network was used for the detection of diabetic retinopathy [32]. The neural network had 28 convolutional layers and, after each layer, there was a batch normalization and ReLU nonlinear function except for the final layer. The MobileNets training was performed in TensorFlow with the help of RMSprop and asynchronous gradient descent.

**Table 3:** Model architectures in the included studies (N=20).

Study	DL^a^ parameters	DL hyperparameters
Al-Makhadmeh et al [17]	Deep belief network, trained the features using the Boltzmann machine classifiers by computing the energy consumption of the network	Cross-entropy loss of 0.0178, L1 loss of 0.0187, and L2 loss of 0.025
Ali et al [18]	Ensemble DL model composed of 5 layers: the input layer, 3 hidden layers, and the output layer; fully connected hidden layer with 20 nodes	Ada optimizer used and a learning rate with a value of 0.03; ReLU^b^ activation function
Dami et al [19]	A deep belief network selected and represented a set of features from the hybrid feature vector and then passed it to the LSTM^c^ neural network. The LSTM neural network consists of 5 layers, including input layers, a hidden layer (with 100 hidden units), 2 fully connected layers, a softmax layer, and an output layer	SGD^d^ for optimizing cross-entropy as the default loss function
Deperlioglu et al [20]	Autoencoder neural network with a hidden layer size of 10. Softmax layer was used	Scaled conjugate gradient algorithm and cross-entropy cost function was used in the coding layer. The coefficient for the L2 weight *regularizer* was 0.001, the coefficient for the sparsity regularization term was 4, and the sparsity proportion was 0.05
Fu et al [21]	A hybrid of CNN^e^ and RNN^f^; 32 convolutional layers (input for CNN) grouped into 8 stages, where each stage was a cascade of four 1D convolutional layers with a kernel size of 16. The final prediction layer was a fully connected dense layer	Before each convolutional layer, a nonlinear transformation occurs, which is a combination of batch normalization, ReLU activation, and a dropout
Huda et al [22]	1D convolution (CNN), max-pooling, and batch normalization. The flattened layer output was passed through a fully connected layer and a second fully connected dense layer. In addition, a softmax layer with 14 outputs was used	Used dropout layers
Torres-Soto et al [23]	Convolutional and pooling layers in the encoder and upsampling and convolutional layers in the decoder; 3 convolutional layers and 3 pooling layers for the encoder segment, and 3 convolutional layers and 3 upsampling layers for the decoder segment of the CDAE^g^	Weights were randomly initiated according to He distribution, and Adam was used as the optimization method. Each model was trained with MSE^h^ loss for 200 epochs, with a reduction in learning rate of 0.001 for every 25 epochs if the validation loss did not improve
Cappon et al [24]	A bidirectional LSTM input layer composed of 128 cells having a look-back period of 15 minutes (ie, 3 samples); 2 LSTM layers composed of 64 and 32 cells, respectively; and a fully connected layer consisting of a single neuron computing the BG^i^ level prediction at 2 different PHs^j^ (ie, 30 and 60 minutes)	BLSTM^k^ architecture, hyperparameters, and look-back period were chosen by trial and error to compromise between model complexity and accuracy
Chen et al [25]	A 3-layered DRNN^l^ with 32 cells in each layer	1, 2, and 4 dilations implemented for the 3 layers from bottom to top, respectively
Efat et al [26]	RNN	In forward propagation, the sigmoid activation function was applied and, for backpropagation, the margin of error of the output was measured, and the weights were adjusted accordingly
Faruqui et al [27]	LSTM with 5-60 layers and 5-40 number of neurons in the feedforward neural network	Dropout rate of 0.10-0.45. An allowable unit change of 0.01 for the dropout rate parameter and of 1 for the number of neurons in LSTM and feedforward layers was selected. A total of 35 × 55 × 35 = 67,375 combinations were tested before finding the optimal hyperparameters
Goyal et al [28]	Faster R-CNN^m^ with ResNet101, Faster R-CNN with Inception-ResnetV2, Faster R-CNN with InceptionV2, and R-FCN^n^ with ResNet101	For Faster R-CNN, the weight was set for L2 regularizer as 0.0, initializer that generated a truncated normal distribution with SD of 0.01 and batch normalization with decay of 0.9997 and epsilon of 0.001. For training, a batch size of 2 was used, optimizer as momentum with manual step learning rate and an initial rate of 0.0002, 0.00002 at epoch 40, and 0.000002 at epoch 60. The momentum optimizer value was set at 0.9. For training R-FCN, the same hyperparameters were used as with Faster R-CNN with the only change being in the learning rate set as 0.0005
Joshi et al [29]	DNN^o^ with 10 hidden layers	Sigmoid activation functions
Sánchez-Delacruz et al [30]	23 assembled algorithms were tested by combining them with the deep RNA multilayer perceptron. The best results were obtained with the combination of FilteredClassifier and the DL model	The base function was applied to the input values, and the softmax was used as the activation function
Sevil et al [31]	Combination of different layers, including fully connected, LSTM, softmax, regression, ReLU, and dropout layers	L2 regularization=0.05
Suriyal et al [32]	MobileNet CNN with 28 layers. The first layer was a fully connected layer	After each layer, there was batch normalization and a ReLU nonlinear function except at the final layer. Training was done in TensorFlow with the help of RMSprop and asynchronous gradient descent
Ech-Cherif et al [33]	Used the MobileNetV2 model, excluded the classification layer, and replaced it with a dense layer that has two classes: benign and malignant	Used pretrained model MobileNetV2. Adam optimizer was used with a starting learning rate of 0.4. For each experiment, the learning rate was decayed by half every 2 epochs. All experiments were run for 55 epochs. Selected batch size was 32
Guo et al [34]	4 sequentially connected convolutional blocks followed by 2 fully connected layers and softmax for the last layer	N/A^p^
Hu et al [35]	ResNet-50 architecture	Number of iterations and batch size optimization were mean average precision and validation classification loss
Uthoff et al [36]	4 sequentially connected convolutional blocks followed by 2 fully connected layers	N/A

^a^DL: deep learning.

^b^ReLU: Rectified Linear Unit.

^c^LSTM: long short-term memory.

^d^SGD: stochastic gradient descent.

^e^CNN: convolutional neural network.

^f^RNN: recurrent neural network.

^g^CDAE: convolutional denoising autoencoder.

^h^MSE: mean squared error.

^i^BG: blood glucose.

^j^PH: prediction horizon.

^k^BLSTM: bidirectional long short-term memory.

^l^DRNN: dilated recurrent neural network.

^m^R-CNN: region-based convolutional neural network.

^n^R-FCN: region-based fully convolutional network.

^o^DNN: deep neural network.

^p^N/A: not applicable.

### Comparison With Classic ML Algorithms

Herein, a presentation of how the DL algorithms used compare with classic ML algorithms, as reported in some of the included studies, is provided. This comparison primarily aims to show whether DL models could bring significant performance gains, which could be critical for their wide adoption by health care providers in routine clinical practice.

In the work by Ali et al [18], the feedforward network for the detection of heart disease based on medical record data and physiological measurements was compared with support vector machine (SVM), random forest, decision tree, and naïve Bayes. The feedforward network achieved 84% accuracy, which was substantially better than the accuracy of classic ML algorithms (72%-80%).

In the work by Dami et al [19], the combination of a deep belief network with LSTM was able to reach 88% accuracy in the prediction of cardiovascular events on data from 4 databases, whereas classic ML algorithms such as logistic regression, SVM, and random forest achieved 56% accuracy on average.

In the paper by Deperlioglu et al [20], AEN was compared thoroughly with additional ML algorithms in other studies. For the PASCAL data set, AEN performed better than all other ML algorithms it was compared with, such as artificial neural networks (82.80-86.50 accuracy), CNN (97.9 accuracy), SVM (90.50 accuracy), naïve Bayes (93.33 accuracy), decision tree (72.76 accuracy), and others. For the PhysioNet data set, AEN performed better than all other ML algorithms, such as CNN (79.50-97.21 accuracy), SVM (83.00 accuracy), wavelet entropy (77.00 accuracy), deep-gated RNA (55.00 accuracy), and others.

DeepBeat in the work by Torres-Soto et al [23] was compared with random forest. However, the sensitivity using random forest was 0.32, the specificity was 0.79, and the *F*_1_ score was 0.39 versus 0.98 sensitivity, 0.99 specificity, and 0.96 *F*_1_ score for the proposed DL methodology.

In the work by Faruqui et al [27], the forecast of daily blood glucose levels through LSTM was achieved with a maximum accuracy of >86% in comparison with the 56% accuracy of k-nearest neighbor regression.

In the work by Goyal et al [28], the localization of DFUs based on imaging data through Faster region-based CNN had a 91.8% average precision. The application of SVM was able to achieve only a 70.3% precision.

Joshi et al [29] applied Levenberg–Marquardt Backpropagation for blood glucose monitoring and achieved an average error of 6.09% in the detection of serum glucose values through near-infrared spectroscopy. However, the use of multiple polynomial regression resulted in a significantly lower average error of 4.88%. This was the only study that showed that a classic ML approach was better than a DL approach.

In the work by Sevil et al [31], the authors compared the performance of their recurrent neural network with k-nearest neighbor, regression SVMs, decision trees, naïve Bayes, Gaussian process regression, ensemble learning, and linear discrimination and regression, which achieved 75.7% to 93.1% accuracy, whereas the proposed approach achieved a classification accuracy of 94.8%.

### Explainability Aspects

When developing models for decision support, there is a need to provide transparent and trustworthy models able to produce not only reliable but also explainable predictions [39]. However, a known problem with DL models is that they lack interpretability and explainability, which hinders their wide adoption in clinical practice.

Explainability deals with the implementation of transparency and traceability of statistical black‐box ML methods. Although attempts to tackle problems related to explanation and interpretability have existed for several years now, there has been an exceptional growth in research efforts in the last couple of years [40]. Approaches for explainability include keeping track of how algorithms are used, which features are the most important for predicting the target variable, and how the algorithm used can be improved, thereby providing hints and clues to guide further developments and enabling the detection of erroneous reasoning through techniques of advanced visualization and signal processing. The challenge is hard as explanations should be sound and complete in statistical and causal terms and yet comprehensible to users, subject to decisions.

This difficulty is also demonstrated in the presented works under review in several cases (eg, in the study by Guo et al [34]). The authors visually analyzed the error cases to better understand why the results were wrong. In only 5% (1/20) of the studies [24], the authors exploited Shapley Additive Explanations (ie, a newly developed approach to interpret DL model predictions [41]). Focusing on predicting glucose concentration in type 1 diabetes, the Shapley Additive Explanations identified that high values of continuous glucose measurements resulted in high predicted blood glucose levels and that high insulin negatively affected the model output.

## Discussion

### Principal Findings

This work presented a systematic literature review of the applications of DL in mHealth for three major chronic diseases that pose a significant international burden: CVD, diabetes, and cancer. To the authors’ knowledge, this is the first systematic review of DL in mHealth for these diseases. The principal outcome of this review is that DL approaches have been used effectively for a variety of diagnostic and predictive tasks in mHealth. More specifically, the most common DL outcomes were found to be (1) diagnosis of the patient’s condition for CVDs, (2) prediction of blood glucose levels for diabetes, and (3) early detection of cancer.

CNNs and recurrent neural networks were the DL algorithms used in most studies. It is worth mentioning that CNNs have been successfully applied to deal with not only computer vision medical tasks but also other tasks based on nonimaging data, such as detection of arrhythmia [22,23] or CVD [21]. Overall, the performance of DL approaches was found to be satisfactory considering that >84% accuracy was achieved in most studies.

In comparison with classic ML approaches, DL was found to achieve better performance in almost all studies that reported such comparison outcomes. This finding shows the value and potential of DL in mHealth for realizing highly intelligent mHealth systems and interventions that could significantly improve clinical decision-making processes. Nevertheless, the authors of this paper acknowledge that DL models require more effort compared with ML models for the preprocessing part, especially when the architecture is based on transfer learning, a common method in most of the image-processing architectures.

The diversity of the identified DL models in the mHealth studies confirms that, for the selection of the most appropriate DL architecture, the one-size-fits-all approach does not apply, a finding that has also been indicated in DL reviews for other fields [42,43]. Another remark is that the architectures of the models in the mHealth studies, as well as the methodologies used for training, were not stated in a consistent manner. This renders the comparison of various approaches between works nontrivial for the interested researcher. None of the included studies used guidelines for reporting the development or outcomes of the models, which could have facilitated the assessment and interpretation of their findings [44].

Most of the included studies dealt with the retrospective technical validation of DL approaches. More thorough external validation is required to prove the generalizability of the DL findings considering that only a minority of the DL studies used an unseen external data set for evaluation purposes. Furthermore, no randomized controlled trials or other types of clinical validation studies with intelligent digital health interventions relying on DL approaches were found in this review [45]. In this respect, further work by the research community is needed to develop DL-empowered systems and applications and prove their clinical effectiveness in health care settings within prospective clinical studies.

Although DL was found to be an effective approach in mHealth for chronic diseases, the explainability of DL outcomes has been scarce. It is apparent that future work is required on the explainability of the DL models developed for chronic diseases as only 5% (1/20) of the studies in this review considered this important dimension [24]. Leveraging explainable models would enhance trust in artificial intelligence and help clinicians make informed judgments [46,47], thereby promoting the real-life use of those models in daily clinical practice. Equally important for the developed models is to support their fairness by ensuring that they mitigate inequalities between individuals and groups of individuals, in particular differences in sex or gender, age, ethnicity, income, education, and geography. In the reviewed studies, mitigation of differences was missing in most cases, merely because of the lack of adequate data. However, if DL models are to be used in daily practice, they should also guarantee fairness and universality [48,49].

### Limitations

This review should be interpreted within the context of its limitations. The authors used a limited set of terms for the search of the literature, including keywords such as *DL* and *neural networks*, combined with keywords related to mHealth. Keywords for specific DL algorithms were not used. This might have inadvertently omitted studies that could have contributed to the progress made in DL applications for mHealth. Articles were searched in a limited number of databases (ie, PubMed and Scopus); two of the most widely used databases internationally nonetheless. No hand search was conducted on any studies reported in other reviews or the included studies, and there was no assessment of the interrater reliability between the authors. A meta-analysis was not possible because of the heterogeneity of the included studies. On the basis of the selected inclusion and exclusion criteria, a small number of eligible studies were included and examined in this review, which limits the generalizability of the findings.

### Conclusions

This review found that DL approaches for chronic diseases could be the vehicle for the translation of big mHealth data into useful knowledge about patient health. Nevertheless, to unlock the full potential of DL, the research community needs to move beyond the conduction of retrospective validation studies and provide robust evidence of the added clinical value of DL-based tools in real-life settings compared with standard care.

## Appendix

Multimedia Appendix 1 PRISMA (Preferred Reporting Items for Systematic Reviews and Meta-Analyses) 2009 checklist.

## Abbreviations

AEN: autoencoder neural network
CDAE: convolutional denoising autoencoder
CNN: convolutional neural network
CVD: cardiovascular disease
DFU: diabetic foot ulcer
DL: deep learning
ECG: electrocardiogram
LSTM: long short-term memory
mHealth: mobile health
ML: machine learning
PRISMA: Preferred Reporting Items for Systematic Reviews and Meta-Analyses
ReLU: Rectified Linear Unit
SVM: support vector machine
TRIPOD: Transparent Reporting of a Multivariable Prediction Model for Individual Prognosis or Diagnosis
